# Mitochondrial aspartate aminotransferase ( *maa1* ) inactivation causes glutamate-requiring *glu1* mutation in *Schizosaccharomyces* *pombe*

**DOI:** 10.17912/micropub.biology.001338

**Published:** 2024-10-21

**Authors:** Kenji Kitamura

**Affiliations:** 1 Department of Gene Science, Natural Science Center for Basic Research and Development, Hiroshima University, Higashi-Hiroshima, Hiroshima, Japan; 2 Genome Biotechnology, Graduate School of Integrated Sciences for Life, Hiroshima University, Higashi-Hiroshima, Hiroshima, Japan

## Abstract

Two genomic genes, which rescue ammonium assimilation defect in the glutamate-requiring *Schizosaccharomyces* *pombe glu1* mutant, were identified. The *maa1* , encoding a mitochondrial aspartate aminotransferase, is the causative gene of *glu1* mutation because an inseparable linkage between *maa1* and *glu1* on the chromosome, and also the *glu1* mutant strain has a nonsense mutation within the *maa1* coding region, which is responsible for its defective phenotype. The *yhm2* , a mitochondrial 2-oxoglutarate carrier, was also isolated as a weak multicopy suppressor gene. These findings reiterate the importance of the mitochondria in utilizing the amino acids for cellular nitrogen metabolism.

**Figure 1. Characterization of glu1 mutant:**
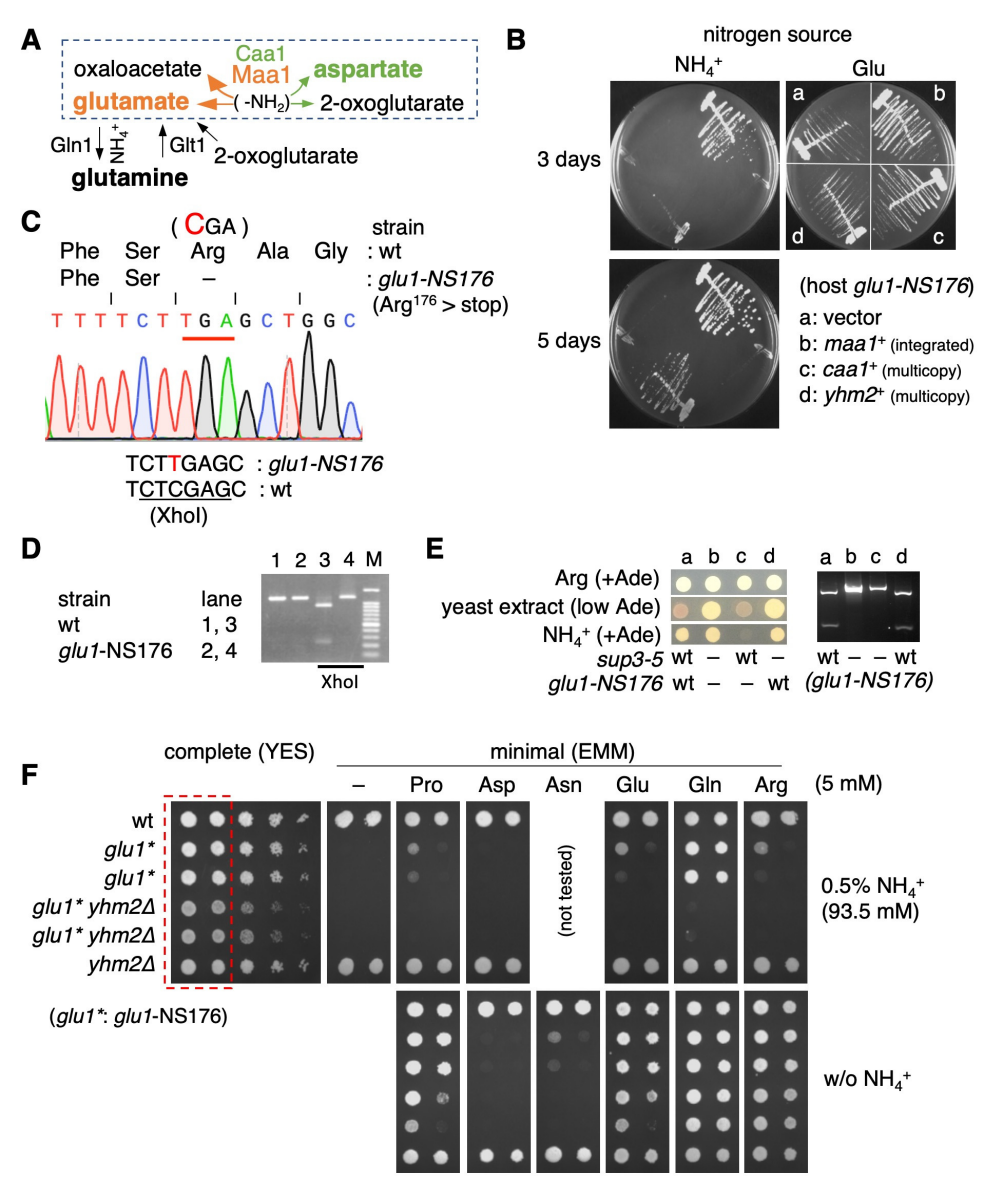
(A) The central role of glutamate in nitrogen metabolism. Glutamate serves as a donor of amino residue in many biosynthetic reactions, and aspartate aminotransferases (Caa1 and Maa1/Glu1) generally catalyze transamination of amino residue from glutamate to oxaloacetate, leading to aspartate synthesis (dotted rectangle). The cytoplasmic Caa1 has a major role for this reaction in *S. pombe* (green). However, mitochondrial Maa1 has a pivotal role in glutamate synthesis by promoting the reaction in an opposite direction (orange). (B) Growth defects in *glu1* mutant were rescued by *maa1* ^+^ or *yhm2* ^+^ . Growth profiles of the *glu1* -NS176 strain (KSP2998) harboring empty vector (a) or plasmid with genomic DNA of either *maa1* ^+^ (b), *caa1* ^+^ (c), or *yhm2* ^+^ (d) *.* Cells were cultured in two nitrogen sources (ammonium or glutamate). The plasmid encoding *maa1* ^+^ gene with its own genomic promoter was integrated into the host chromosome of the *glu1* -NS176 mutant (b, KSP4250), although the plasmids were episomal in other three strains (a, c, d) . Growth defects of *glu1* -NS176 strain in ammonium medium (a, vector) were recovered by *maa1* ^+^ (b) or *yhm2* ^+^ (d) but not by *caa1* ^+^ (c) plasmid. Notably, the effect of *yhm2* ^+^ is weaker than that of *maa1* ^+^ since the *yhm2* ^+^ plasmid-containing *glu1* strain (d) grew slowly than the strain in which the mutation was corrected by the *maa1* ^+^ gene (b). All the strains grew when glutamate was used as the sole nitrogen source (right panel). (C) Nonsense mutation in the *maa1* gene in *glu1-NS176* mutant. The C-to-T change in the mutant gene converts the CGA codon for Arg ^176^ to the opal codon (TGA), producing a truncated, non-functional protein. The sequence of this region in the wild-type strain (CTCGAG) is an XhoI recognition sequence, but the mutation prevents this recognition. (D) Discrimination of *glu1* mutant by XhoI digestion. The genomic *glu1* gene from the wild-type and mutant strains was amplified by PCR. Only the mutant derived-DNA fragment remained unchanged after XhoI digestion (lanes 3 and 4), which can be used for diagnosing this nonsense allele. M: size marker. (E) The *glu1-NS167* mutation is suppressed by the *sup3-5* nonsense suppressor. (Left panel) The cells of the four strains were spotted in the indicated medium. All strains grew when arginine was used as the sole nitrogen source (Arg). All strains had *ade6-704* , an opal nonsense mutation, which is red in low adenine medium (low Ade). The *sup3-5* mutation (indicated by –) suppressed *ade6-704* , thus enabling white colony formation (strains b and d). Both strains b and c had *glu1-NS167* mutation, but only strain c showed growth defects in the ammonium medium because of the absence of *sup3-5* mutation (wt) in this strain. (Right panel) Diagnosis by XhoI digestion confirmed that strains b and c are *glu1* mutants (shown as –), and strains a and d are wild-type. Strains a, b, c, and d represent KSP4263, KSP4264, KSP4265, and KSP4266, respectively. (F) Serially diluted cells of the indicated strains were spotted on each medium and grown for two days in YES medium or three days in EMM. The amino acids were added at 5 mM. For the EMM results, only the spots of the highest and second highest cell concentrations, corresponding to the dotted rectangle in the YES medium, are shown. All the strains used in this figure were prototrophic, except for *glu1* mutation. Strains: wt (L972); *glu1* (KSP4267, KSP4268); *glu1 yhm2* (KSP4269, KSP4270); *yhm2* (KSP4247).

## Description

Glutamate plays a central role in cellular nitrogen metabolism (Magasanik 2003, Lijungdahl and Daignan-Fornier 2012) . It accepts free ammonium to form glutamine by glutamine synthetase ( Gln1 ) or confers its amino residue as a donor to other 2-oxo acids by various aminotransferases ( Figure 1A ). The *glu1* (glutamate-requiring) mutant (Kohli et al., 1977) has been previously used as an auxotroph to monitor oligopeptide utilization by *Schizosaccharomyces pombe* cells (Kitamura et al., 2012) . This mutant cannot grow in standard minimal medium (EMM) with ammonium as a nitrogen source and has unique amino acid availability traits. The *glu1* mutant grew when glutamate was used as the sole nitrogen source, although glutamate poorly supported its growth in the presence of ammonium (Figures 1B and F). Interestingly, glutamine efficiently rescues the growth defect of the *glu1* mutant compared to glutamate, even in the presence of ammonium ( Figure 1F, B arel and MacDonald 1993). Additionally, proline and arginine weakly supports *glu1* strain growth. In general, their availability is regulated by nitrogen catabolite repression; thus, it is greatly affected by the presence of ammonium, a high-quality nitrogen source. The *glu1* mutant grew normally when proline or arginine was used as the sole nitrogen source ( Figure 1F ). These traits indicated that *glu1* mutant had a defective nitrogen metabolism.

Possibly because of this arginine-mediated growth recovery, *glu1* was once described to be allelic to an *arg* gene (Kohli et al., 1977) . Classical genetic chromosomal mapping shows that both *glu1* and *arg6* are closely linked to *ade7* on chromosome 2 (Kohli et al., 1977) . Interestingly, the *arg6 ^+^* gene-harboring plasmid failed to rescue the *glu1* -NS176 mutant (our unpublished data). Since the molecular entity of the *glu1* gene has not been demonstrated so far, a gene supporting the *glu1* -NS176 strain growth in ammonium medium was isolated from the *S. pombe* genomic library. In this screening, the *maa1* gene (SPBC725.01), which encodes mitochondrial aspartate aminotransferase, was identified. It catalyzes the transamination between oxaloacetate and glutamate, leading to the formation of aspartate and 2-oxoglutarate ( Figure 1A ). Since this reaction can proceed in both directions, the same enzyme also supplies glutamate by catalyzing the reverse reaction. We concluded that *glu1* was identical to the *maa1* by following four pieces of evidence: 1) Even the single-copy *maa1 ^+^* gene efficiently rescues the *glu1* -NS176 mutation by chromosomal integration ( Figure 1B ). 2) Chromosomally integrated *maa1 ^+^* is tightly linked to the original *glu1* mutation. No recombination events occurred between chromosomally integrated *maa1 ^+^* and *glu1* -NS176 mutation in descendant 84 spores from 22 asci according to tetrad analysis. 3) The *glu1* -NS176 allele harbors a C-to-T mutation in the *maa1* coding region. Although the ambiguity of this mutant allele among *glu1* mutants described previously (Barel and MacDonald 1993) , we refer this mutant allele as *glu1* -NS176 since this mutation converts the CGA codon corresponding to the 176th arginine to the TGA ^opal^ **n** on **s** ense codon ( Figure 1C ). The wild-type Maa1 protein is 437 amino acids long, but this nonsense mutant allele produces a C-terminally truncated protein with the first 175 amino acids only. Because the amino acid sequence of the missing region of the mutant protein is well conserved in aminotransferase family proteins, this truncated protein is supposed to be non-functional. The occurrence of the nonsense codon disrupts the XhoI recognition site in the corresponding sequence ( Figure 1C ), thus XhoI digestion can discriminate between wild-type and mutant alleles ( Figure 1D ). 4) The *glu1* -NS176 mutation was rescued by *sup3* -5 ( Figure 1E ), a tRNA ^Ser^ (SPATRNASER.03) suppressor mutation that suppresses the TGA ^opal^ nonsense codon (Hottinger et al., 1982, Niwa et al., 1989) . This is direct proof that the observed growth defect in the ammonium medium is caused by the *maa1* gene nonsense mutation in the *glu1* -NS176 strain. Although the *maa1* ∆ mutant has not been examined in detail (Reidman et al., 2019) , its characteristics are similar to those of the non-functional *glu1-NS176* mutant.

*S. pombe* harbors the cytoplasmic aspartate aminotransferase gene *caa1* (Reidman et al., 2019) . Multicopy plasmid-borne *caa1 ^+^* failed to rescue the growth defect of the *glu1* -NS176 mutant in ammonium media ( Figure 1B ), unlike the effect of the mitochondrial enzyme *glu1* / *maa1* . Since using aspartate as a nitrogen source cannot support *glu1* mutant growth ( Figure 1F ), growth defects in this mutant may be related to glutamate, but not aspartate, shortage. In addition to *glu1* / *maa1* , the *yhm2* gene (SPBC83.13) is a multicopy weak suppressor of the growth defect in the *glu1* -NS176 mutant. The *glu1* mutant harboring the multicopy *yhm2* ^+^ plasmid grew slower than the *glu1* -corrected, wild-type strain ( *maa1* ^+^ (integrated); Figure 1B ). The Yhm2 homolog in *Saccharomyces cerevisiae* is a mitochondrial carrier protein that imports 2-oxoglutarate into the mitochondria (Castegna et al., 2010) . Therefore, increasing the 2-oxoglutarate concentration in mitochondria may alleviate the ammonium utilization defect in the *glu1* -NS176 mutant. The *yhm2* ∆ strain showed no growth defects under all medium conditions examined, whereas the *glu1* and *yhm2* ∆ mutations had an additive effect. The *glu1* -NS176 *yhm2* ∆ double mutant grew slower than each single mutant in yeast extract medium ( Figure 1F ). The *S. pombe glu1* -NS176 *yhm2* ∆ double mutant grew when amino acids, except aspartate and asparagine, were used as the sole nitrogen source ( Figure 1F ). Interestingly, all the tested amino acids, including glutamine, did not support the growth of double mutant in the presence of ammonium. *S. cerevisiae* mutants, which lack all mitochondrial 2-oxoglutarate carriers including Yhm2 , cannot grow when ammonium is the sole nitrogen source, and importantly, this growth defect is rescued by the addition of glutamate (Palmieri et al., 2001, Castegna et al., 2010, Scarcia et al., 2017) .

Proline is metabolized to glutamate before use as a nitrogen source. The *glu1* strain can grow well by supplying proline or glutamate in the absence of a preferable nitrogen source, contrasting to the situation in the *glu1 yhm2∆* double mutant whose growth is weak in this condition. In *S. cerevisiae* , Yhm2 , Odc1, and Odc2 function complementarily as mitochondrial 2-oxoglutarate carriers (Scarcia et al., 2017) , and Yhm2 is reported to be involved in the shuttle of NADPH (reducing power) by transporting 2-oxoglutarate and citrate in exchange across the mitochondrial membrane (Castegna et al., 2010) . Since glutamate and 2-oxoglutarate are closely related, it is possible that *glu1* mutation in the absence of Yhm2 ( *yhm2* ∆), where 2-oxoglutarate transport is reduced, may cause problems with metabolism of glutamate and 2-oxoglutarate, their turnover, and NADPH redox balance. We assume that the growth of the *glu1 yhm2* ∆ double mutant is attenuated as the sum of these abnormalities.

Two genes encode aspartate aminotransferase in *S. pombe* , but the phenotypes of each mutant are very different. The *caa1* mutant is auxotrophic for aspartate (Reidman et al., 2019) . In contrast, glutamate or glutamine, but not aspartate, is necessary for growth of the *glu1* mutant ( Figure 1F ). Thus, Caa1 and Maa1 /Glu1 have independent, non-overlapping roles and mainly catalyze aspartate and glutamate synthesis, respectively. Unlike glutamate, glutamine supported *glu1* mutant growth very effectively, even in the presence of ammonium ( Figure 1F ), whereas high-dosage expression of the glutamine synthetase *gln1* ^+^ from the multicopy plasmid did not (our unpublished data). It is possible that the 2-oxoglutarate conversion to glutamate by Maa1 is limited in the *glu1* mutant, and in turn, glutamate shortage interferes with its use as a glutamine synthetase ( Gln1 ) substrate. The effect of inactivating Glt1 , a glutamate synthase (Sasaki et al., 2017) , was also examined, but the phenotype of *glu1 glt1* ∆ double mutant was indistinguishable from that of the parental *glu1* strain (our unpublished data). Collectively, glutamate shortage is likely the major cause of the ammonium assimilation defect in the *glu1* mutant.

The *glu1* mutant was originally reported as one of the four glutamate-requiring mutants ( *glu1* – *glu4* ). Investigating these mutants revealed defects in specific mitochondrial enzymatic activities, except *glu1* (Barel and MacDonald 1993) . These genes are registered in PomBase (Rutherford et al., 2024) : NAD ^+^ -dependent isocitrate dehydrogenase (IDH) subunit 2 ( *glu2* / *idh2* /SPBC902.05c), subunit 1 ( *glu3* / *idh1* /SPAC11G7.03), and glutamate synthase ( *glu4* / *glt1* /SPAPB1E7.07). Together with the finding of *glu1* / *maa1* in this study, all four *glu* genes and *yhm2* (2-oxoglutarate carrier) are related to mitochondria, reiterating the importance of mitochondria in cellular nitrogen metabolism.

## Methods

*S. pombe* media and genetic procedures

The composition of yeast media and other molecular methods have been described previously (Rhind and Forsburg 2006). When specified, the NH _4_ Cl in the EMM was replaced with 5 mM amino acids as the nitrogen source.

Isolation of *S. pombe* genes rescuing *glu1* growth defect

The *glu1* host strain (KSP2998) was transformed using the *S. pombe* genomic library (obtained from YGRC, NBRP), and colonies growing on ammonium medium without other nitrogen sources were selected. Plasmids were recovered, and the nucleotide sequences of both ends of the inserted genomic DNA were determined. Using these sequences as queries, a BLAST search was performed against the genome data in PomBase (Wood et al., 2012, Rutherford et al., 2024) to identify the chromosome-derived region in the plasmid. Three plasmids for *maa1* and six plasmids for *yhm2* were recovered from nine independent *S. pombe* colonies.

*glu1* -NS176 sequence determination and discrimination by restriction endonuclease

The *maa1* / *glu1* gene from the *glu1* mutant (KSP2998) was obtained by PCR using Quick Taq HS DyeMix (Toyobo, Japan) and primers (glu1-1 and glu1-3). The nucleotide sequence of the amplified DNA was determined and compared with the genome data from PomBase. To discriminate between wild-type and mutant strains, amplified DNA with primers (glu1 *-2* and glu1 *-3* ) from test strains was treated with XhoI and analyzed by electrophoresis.

## Reagents

Yeast strains

**Table:** 

name	relevant genotype	source
FY8752	*glu1* *ade7 ura5*	*YGRC
FY23947	*yhm2 ::kanMX ade6-M21x ura4-D18 leu1-32 *	*YGRC
HM518	*ade6-704 Ch10-CN2(dg::sup3-5) leu1-32*	Dr. O Niwa
KSP2998	*glu1-NS176 leu1-32*	this study
KSP4247	*yhm2 ::kanMX *	this study
KSP4250	* glu1-NS176::( maa1 ^+^ , LEU2 ^Sc^ ) leu1-32 *	this study
KSP4263	*ade6-704 leu1-32*	this study
KSP4264	*glu1-NS176 ade6-704 leu1-32 Ch10-CN2(dg::sup3-5)*	this study
KSP4265	*glu1-NS176 ade6-704 leu1-32*	this study
KSP4266	*ade6-704 leu1-32 Ch10-CN2(dg::sup3-5)*	this study
KSP4267	*glu1-NS176*	this study
KSP4268	*glu1-NS176*	this study
KSP4269	* glu1-NS176 yhm2 ::kanMX *	this study
KSP4270	* glu1-NS176 yhm2 ::kanMX *	this study
L972	wild type (prototroph)	our stock

*YGRC: Yeast Genetic Resource Center

Oligonucleotide primers

**Table:** 

glu1-1	5′-CGAGTTACTGTTTACTAGTTGC-3′
glu1-2	5′-GCTGTTGAGTCAGAAACTCGAC-3′
glu1-3	5′-GTGTGTTTGAGTCTGTCTATATGTATG-3′
